# Beyond Charge Transfer: The Impact of Auger Recombination and FRET on PL Quenching in an rGO-QDs System

**DOI:** 10.3390/nano11061623

**Published:** 2021-06-21

**Authors:** Anton A. Babaev, Anastasiia V. Sokolova, Sergei A. Cherevkov, Kevin Berwick, Alexander V. Baranov, Anatoly V. Fedorov, Aleksandr P. Litvin

**Affiliations:** 1 Center of Information Optical Technology, ITMO University, 197101 St. Petersburg, Russia; avsokolova@itmo.ru (A.V.S.); s.cherevkov@itmo.ru (S.A.C.); a_v_baranov@itmo.ru (A.V.B.); a_v_fedorov@itmo.ru (A.V.F.); litvin@itmo.ru (A.P.L.); 2 School of Electrical and Electronic Engineering, TU Dublin, Grangegorman, Dublin 7, Ireland; kevin.berwick@tudublin.ie

**Keywords:** quantum dots, reduced graphene oxide, spectroscopy, PL lifetime, Auger recombination

## Abstract

PL intensity quenching and the PL lifetime reduction of fluorophores located close to graphene derivatives are generally explained by charge and energy transfer processes. Analyzing the PL from PbS QDs in rGO/QD systems, we observed a substantial reduction in average PL lifetimes with an increase in rGO content that cannot be interpreted solely by these two processes. To explain the PL lifetime dependence on the rGO/QD component ratio, we propose a model based on the Auger recombination of excitations involving excess holes left in the QDs after the charge transfer process. To validate the model, we conducted additional experiments involving the external engineering of free charge carriers, which confirmed the role of excess holes as the main QD PL quenching source. A mathematical simulation of the model demonstrated that the energy transfer between neighboring QDs must also be considered to explain the experimental data carefully. Together, Auger recombination and energy transfer simulation offers us an excellent fit for the average PL lifetime dependence on the component ratio of the rGO/QD system.

## 1. Introduction

Since the beginning of the 21st century, colloidal quantum dots (QDs) have been one of the most promising materials for photonic applications [1,2,3,4,5,6] due to their unique physical properties, together with the availability of a wide variety of processing methods. QDs are versatile building blocks with highly tunable optical and electrical properties that can be easily modified by varying their size and chemical composition during synthesis and post-synthesis treatment [7,8,9,10]. As nanocomposites and nanostructures, QDs have many applications, replacing traditional material systems.

QD/graphene systems are a type of novel nanostructure with advanced properties that can potentially be used for optoelectronic applications [11,12]. QD/graphene composites can be applied as a top layer in a solar cell to significantly enhance the device’s stability by reducing moisture ingress [13]. It was also shown that graphene derivatives with suitable treatment can serve as materials that are transparent and highly conductive with a narrow bandgap. These materials can improve the morphology and charge transport properties of QD films in order to improve solar cell performance [14]. Both studies mentioned used the same approach to QD/graphene system formation, viz. a reduced graphene oxide (rGO) functionalization by an organic linker (3-mercaptopropyl) trimethoxysilane (MPTS), first reported in 2015 [15]. Typically, the interaction between QDs and graphene derivatives in these nanostructures leads to QD photoluminescence (PL) quenching [15,16,17,18,19,20,21]. This behavior is usually described by simple models that consider only charge carrier transfer and nonradiative energy transfer from QDs to the graphene derivative. Moreover, the contribution from nonradiative energy transfer is often neglected, which allows the estimation of the charge transfer rate by analyzing the PL lifetimes of pristine QDs and QD/graphene nanostructures [15,16,22,23].

However, QDs possess intrinsic features that can significantly affect the observed PL decay. The blinking effect in QDs is one example and can dramatically reduce the PL lifetime [24]. The main mechanism responsible for blinking is excess charge carriers in QDs that induce an electric field, triggering non-radiative Auger recombination. Fast relaxation through this channel competes with radiative relaxation, leading to quenching of the QD PL intensity and PL lifetime reduction [25]. In the theory describing the blinking effect, the pathway of QD ionization is debatable, whereas in QD-based nanostructures, charging of QDs can occur during charge transfer.

In this paper, we study the PL kinetics in a system of closely packed PbS QDs covalently attached to the rGO surface by a MPTS linker. We demonstrate that Auger relaxation must be considered as well as PL quenching in QD/graphene systems. A thorough analysis of the steady-state and kinetic PL properties of PbS QDs in QD/rGO systems with different QD/rGO ratios demonstrates that the variation in QD PL properties cannot be explained by a simple charge transfer model. We demonstrated that an Auger recombination induced by residual holes dominates the excitation relaxation in these systems. We believe that the model proposed here is necessary for the correct analysis and interpretation of QD PL in complex nanostructures.

## 2. Materials and Methods

Precursors for QDs synthesis PbO (99.999%), hexamethyldisilathiane, (99.99%) were purchased from Sigma-Aldrich (Saint Louis, MO, USA); octadecene (ODE) was obtained from Acros (Geel, Belgium); oleic acid from Fisher Chemicals (Waltham, MA, USA); EtOH (ethanol), MeOH (methanol), and chloroform were purchased from Vekton (St. Petersburg, Russia). The 95% (3-Mercaptopropyl) trimethoxysilane (MPTS), reduced graphene oxide (rGO) (Merck (777684)), n–methylformamide (NMF) was purchased from Sigma-Aldrich (Saint Louis, MO, USA).

PbS QDs with a diameter of 3.6 nm were synthesized using a hot injection method [26,27]. The synthesized QDs had an absorption peak at a wavelength of 1000 nm and a PL peak at 1090 nm (Figure S1). The method adapted from [15] was used for rGO functionalization. In brief, rGO powder was dissolved in NMF by ultrasonication for 1 h. The concentration of the rGO solution obtained was 1 mg/mL. Then, 250 μL of MPTS was added to 1 mL of rGO solution, followed by ultrasonication under an argon atmosphere for 16 h at 40 °C. The solution was then purified by centrifugation with an excess of ethanol to remove the unreacted products.

The used rGO had >75 wt % of carbon; <22 wt % of oxygen; and epoxy, carboxy, and hydroxy functional groups. The MPTS molecule had a thiol group on one side and a silane group on another side. The silane group covalently binds with the specified functional groups on the rGO surface, as was previously shown [15,28,29,30,31]. The free thiol group on another side of the MPTS molecule remains free to be attached to the QD surface [15]. The FTIR spectra after the functionalization are shown in Figure S2b. The peaks at 752, 1091, and 1247 correspond to –CH_2_–CH_2_–, Si–O–C, and Si–C bonds, respectively, and are typical of MPTS [32]. The size of rGO flakes lies in the range from hundreds of nm to tens of µm, as shown in the electron microscopy analysis in Figure S3.

The QD-rGO systems were obtained by vortexing the functionalized rGO and QD solutions at 2000 rpm for 5 min. To separate unbound QDs, the solution was precipitated by centrifugation. The supernatant was discarded, and the precipitate was dissolved in NMF. After the second purification, the precipitate was dissolved in chloroform.

Absorption spectra were measured using a Shimadzu UV3600 spectrophotometer (Shimadzu corporation, Kyoto, Japan). Photoluminescence (PL) spectra and PL decay curves were measured using a custom-built setup for the NIR PL analysis, which is described in more detail in [33,34,35]. For the PL decay measurements, a 532 nm Nd:YAG laser with a 1 ns pulse duration was used for excitation, and an InGaAs/InP avalanche photodiode (APD) operating in a photon-counting mode was used for light detection. For the PL spectra measurements, a 633 nm He-Ne laser was used for excitation, and an InGaAs photodiode was used for detection.

## 3. Results

**Figure 1:**
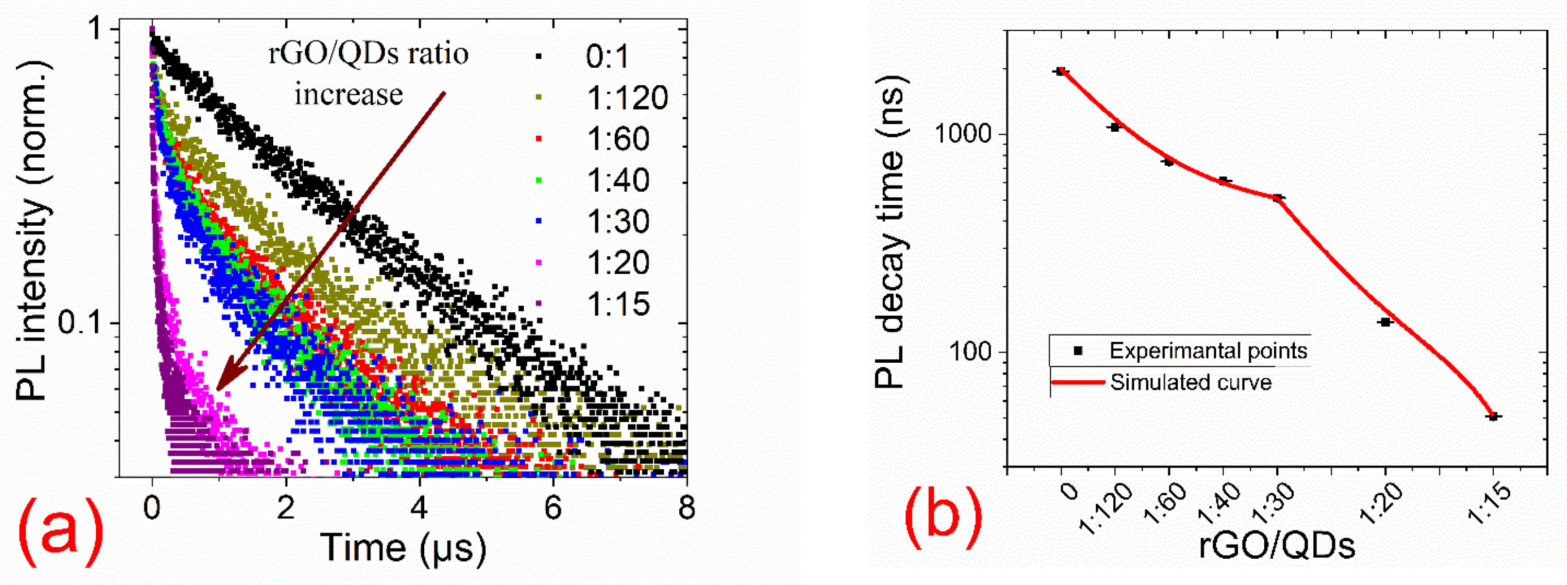
(**a**) PL decay curves of QD-rGO systems with various rGO/QD ratios; (**b**) average PL lifetimes for the QD-rGO systems plotted vs. rGO/QD ratio together with the curve calculated assuming that PL quenching is caused by Auger recombination and energy transfer.

The PbS QDs-rGO solutions were prepared with rGO to QDs weight ratios of 1:120, 1:60, 1:40, 1:30, 1:20, and 1:15, at which the close packing of QDs is expected [15]. The pure PbS QDs solution was used as a reference. First, we investigated the dependence of QD PL lifetime on the rGO/QD ratio. The PL decay curves recorded for solutions with various rGO/QD ratios are shown in Figure 1a. The decay for the reference solution was fitted by a biexponential function, and decays for the rGO/QD solutions were fitted by a triexponential function. The average PL lifetime was calculated using the following equation:

(1) $$\tau_{\mathrm{av}}=\frac{\sum_{i}A_{i}t_{i}}{\sum_{i}A_{i}},$$

where $A_{i}$ and $t_{i}$ are the amplitudes and times extracted by multiexponential fitting of the decay curve, respectively. The calculated average PL lifetimes are listed in Table 1 and plotted in Figure 1b.

From Figure 1a and Table 1, PL lifetimes dramatically reduce with an increase in the rGO/QD ratio. The τ_av_(rGO/QD) dependence possesses a complicated form. Varying the rGO/QD ratio from 1:120 to 1:30, we observed a halving of PL lifetime, whereas changing the rGO/QD ratio from 1:30 to 1:15 reduced the PL lifetime by a factor of 10. Assuming that the change in PL lifetimes is caused entirely by charge and energy transfers from QDs to rGO, we must assume a strong enhancement in these processes. Enhancement in the energy and charge transfers may be due to the reduction in the distance between the nanoparticles, or it may be due to an increasing number of quenchers. The distance between the QDs and rGO is constant and equal to the length of the MPTS linker. The increase in the number of quenchers (rGO) for this system suggests the connection of a single QD to multiple rGO sheets. However, the attachment of a QD to more than two rGO sheets is impossible due to the geometric parameters of these nanostructures. Since the unreacted products and unbounded QDs were removed from the solution, the diffusion-controlled PL quenching could not appreciably contribute to the results of the measurements. Therefore, increasing the number of quenchers cannot explain the gradual reduction in QD PL lifetimes.

It is known that a hopping-like charge transfer is dependent on the number of unoccupied states in an acceptor [36]. Therefore, without effective electron withdrawal from the rGO sheets, the electron transfer should saturate when all free rGO electron states lying lower than the QD lowest unoccupied molecular orbital (LUMO) level are filled by electrons from QDs. These conditions are reasonable for the rGO-QD system since electron transfer and storage on rGO have been previously observed [16,17,37]. In this case, the dependence of PL lifetime on the rGO to QD ratio may be driven by some QD-independent rGO discharging processes, such as electron scavenging by O_2_ molecules. The rate of this discharging process would be expected to be the same for all rGO to QD ratios. The freed electron states would be filled by electrons from QDs, leading to quenching of the PL from a constant number of QDs. A higher rGO to QD ratio would result in a higher quenched/unquenched QD ratio and shorter PL lifetimes. The expected plot of $\tau_{av}$ to rGO/QD ratio is easy to simulate, assuming the PL decay law can be described as:

(2) $$I\left(t\right)=\sum_{i}a_{i}e^{-\frac{t}{\tau_{i}}},$$

where $a_{i}$ are the relative PL amplitudes of the QDs, normalized to unity with PL decay time $\tau_{i}$ . We performed this calculation, and the results are provided in Figure S4 (Supplementary Materials). From Figure S4, the average PL lifetime is dominated by high QY PL from unquenched QDs. The reduction in the average PL lifetime was observed only in low unquenched/quenched QD ratios, and the resulting curve does not fit the experimental data well.

**Scheme 1:**
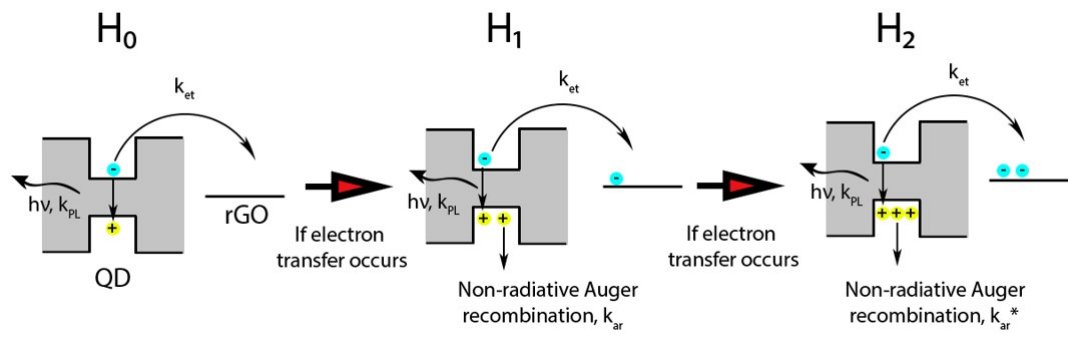
Possible QD exciton relaxation pathways in the QD-rGO system. The circles with + sign depict the holes, the circles with − sign depict the electrons, the * after the $k_{\mathrm{ar}}$ is to highlight the different rates in case of 2 excess holes.

To explain the excitation relaxation in QDs located near the rGO surface, we needed to carefully analyze the PL dynamics in QDs during charge transfer to the rGO sheets. We formed several assumptions: (1) we did not consider the electric field, which can arise from the accumulation of charge carriers on rGO sheets; (2) we assumed that the transfer of charge carriers does not lead to a change in the rGO oxidation level because of the high carbon content (>95%); (3) we neglected nonradiative energy transfer from QDs to rGO since its efficiency is insignificant [22]; (4) we assumed that the transfer of holes from QDs to the rGO is negligible compared to electron transfer [16,17,37]. With these assumptions, we predicted several possible configurations in which QDs can exist in a QD-rGO system coupled with a charge transfer process (Scheme 1). If the QD in a QD-rGO system is electrically neutral (identified as the H_0_ state for 0 excess holes), the exciton formed in the QD has several relaxation pathways. These rates include k_r_, k_et_, and k_nr_, where k_r_ is the radiative recombination rate, k_et_ is the charge carrier (electron) transfer rate, and k_nr_ is the nonradiative relaxation rate.

If an electron transfer has occurred, the QD state changes from H_0_ (zero excess holes) to H_1_ (one excess hole). Since there is an excess charge carrier in the H_1_ state, the non-radiative Auger recombination channel with rate k_A_ becomes open for the next exciton formed in this QD. The PL of the QD in the H_1_ state is well-described by the theory of QD blinking [25,38,39]. The k_r_ rate is typically much lower than k_A,_ resulting in strong PL intensity quenching and a significant reduction in PL decay time. The QD stays in the H_1_ state until one of the following two processes occurs: Firstly, transferring an excess hole from a QD to rGO leads to the conversion the QD back to an H_0_ state (as mentioned above, the efficiency of this process is low) or absorption of the next photon and generation of a new exciton. In the second case, radiative and non-radiative recombination (including Auger recombination) competes with a second electron transfer from a QD to the rGO, which switches the QD to an H_2_ state (two excess holes). The electric field induced by two excess charge carriers resulted in an even more effective non-radiative Auger recombination for the subsequent excitation and, consequently, stronger PL quenching and shortening of the PL decay [39]. Owing to these considerations, it follows that QDs in a QD-rGO system may have typical lifetimes, which are different for the H_0_–H_2_ states and can be described using the blinking model [39]:

(3) $$\tau_{0}=\frac{1}{k_{r}+k_{\mathrm{et}}+k_{\mathrm{nr}}},\;\tau_{1}=\frac{1}{2k_{r}+k_{\mathrm{nr}}+k_{\mathrm{et}}+2k_{A}},\;\tau_{2}=\frac{1}{3k_{r}+k_{\mathrm{nr}}+k_{\mathrm{et}}+6k_{A}},$$

where $k_{A}$ is the Auger recombination rate.

As discussed earlier, without an effective rGO discharging process, the electron transfer should saturate when all free electron states of rGO lying lower than the QDs LUMO are filled by electrons from the QDs. Due to our assumptions, the distribution of excess holes in the QD ensemble should not significantly change between excitation laser pulses. The LUMO of the rGO in the saturated system would be balanced with the QDs LUMO. In this case, electrons transferred from QDs can transfer back to the QDs. Since the $\tau_{i}$ reduces rapidly with charge number (i) growths, the probability of electrons to be transferred from QDs to rGO also reduces since this process competes with radiative and Auger recombination. Thus, we expect: (1) electrons from QDs with a lower charge are more likely to be transferred to the rGO sheet. If the back electron transfer from rGO to QD occurs, this electron can recombine with a hole left in a QD. For the overall system, this means that: (2) electrons trapped by QDs from the rGO conductive band are more likely to recombine in more highly charged QDs. These two processes would drive each QD to have an excess carrier number close to the mean value for an ensemble of QDs.

So, the PL lifetime of systems with integer (i) mean charge number tends to have the same PL lifetime of QDs in the H_i_ state. If the mean charge number is not an integer, the QD population will be distributed between two adjacent states (H_i_ and H_i+1_). The PL decay of this system can be described as:

(4) $$I\left(t\right)=a_{i}e^{-\frac{t}{\tau_{i}}}+a_{i+1}e^{-\frac{t}{\tau_{i+1}}},$$

where $a_{i}$ and $a_{i+1}$ are the relative contributions of QDs, normalized to unity, with i and i + 1 excess charge carriers, respectively. The $a_{i}$ and $a_{i+1}$ coefficients depend on the QDs number ( $N_{i}$ ) as $a_{i}\sim N_{i}Q_{i}$ where $Q_{i}=\tau_{i}k_{\mathrm{PL}}$ is the QY of a QD with i excess charge carriers. The increase in the rGO to QD ratio will proportionally change the $N_{i}$ to $N_{i+1}$ ratio until the excess of charges is sufficient to occupy the H_i + 1_ state for all QDs. However, in practice, PL decay curve fitting usually requires three exponents due to the energy transfer process and complicated energy structure of PbS QDs. So, we can simplify this model using just two exponents, with $\tau_{i}$ and $\tau_{i+1}$ describing the average PL lifetimes of QDs with corresponding excess charge carriers.

In summary, the PL lifetime should shorten when the ratio of rGO/QD increases as the QDs become more charged and $\tau_{0}\gg \tau_{1}\gg \tau_{2}$ . When the mean charge number of the QDs is integer i, the PL lifetime should be close to the PL lifetime of a QD in the H_i_ state. The PL lifetime vs. rGO/QD ratio plot should consist of H_i_ points connected with nonlinear curves.

Analyzing the PL lifetimes on the rGO plot from Figure 1b, we can observe a critical point at the 1:30 ratio. According to our model, this point should appear at the rGO/QD ratio corresponding to all QDs in the H_1_ state. From this, we assume $\tau_{1}$ ≈ 510 ± 5 ns. It is somewhat surprising to find a PL lifetime for the QDs with one excess hole this long. The k_A_ calculated according to Equation (A1) is about 6.84 × 10^5^ s^−1^, which is three orders of magnitude smaller than that observed for single CdSe QDs [39]. However, system conditions in a colloidal solution are vastly different from those for single QDs on a substrate.

To validate our model experimentally, we calculated $\tau_{\mathrm{av}}$ vs. the rGO/QD ratio. The calculation is based on a combination of Förster resonance energy transfer (FRET) between QDs and Auger-induced quenching of the QD PL. FRET is another mechanism that can quench neighboring QDs and lead to an overall reduction in PL lifetimes. FRET between QDs with vastly different PL lifetimes is exceptionally complicated, with uncommon features like delayed fluorescence [40] sometimes present. A detailed description of the simulation algorithm can be found in the Supplementary Materials. The resulting curve is depicted in Figure 1b. Clearly, the simulated curve is an excellent fit for the experimental data.

**Figure 2:**
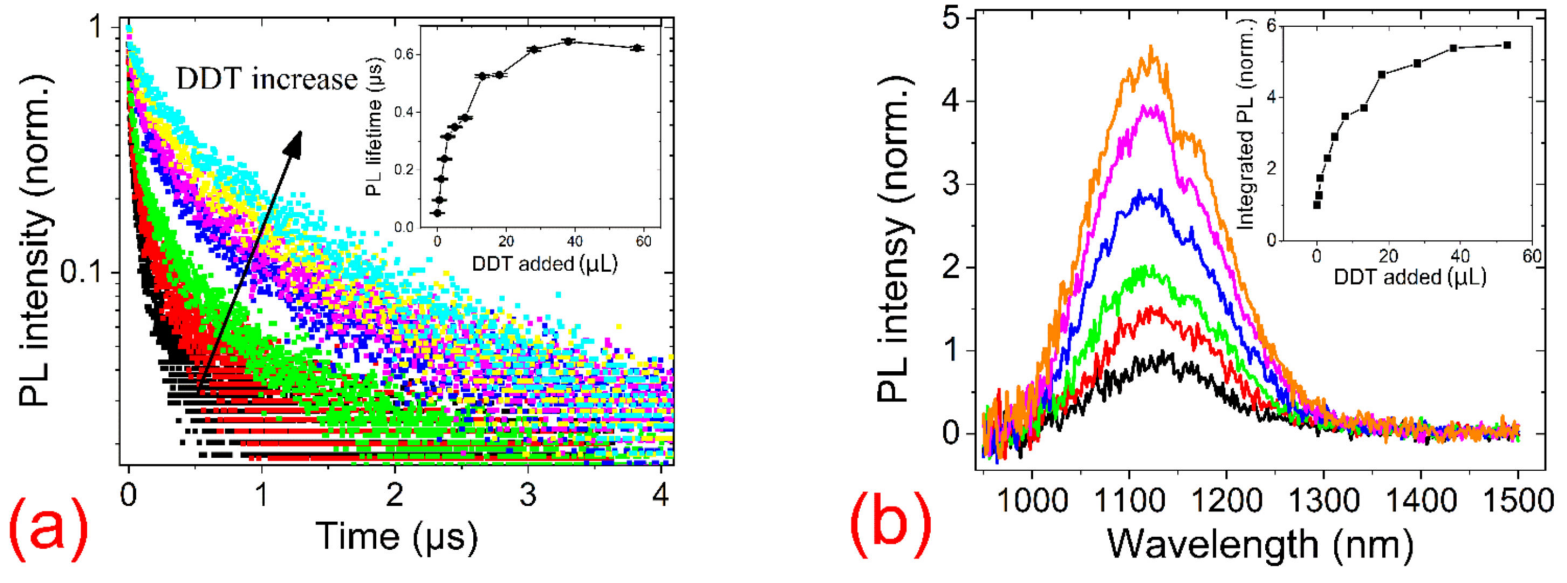
(**a**) PL decay curves for the 1:15 QD-rGO system with different amounts of added DDT. Inset—average PL lifetimes of the 1:15 QD-rGO system plotted vs. amount of added DDT; (**b**) PL spectra for the 1:15 QD-rGO system with different amounts of added DDT. Inset—integrated PL intensity vs. amount of added DDT.

Recombination via an excess of free carriers (holes) is a fundamental element of the proposed model. Experiments involving the engineering of the free carrier density were performed to provide support for this theory. To control the density of free holes, we studied the reference QD solution and the QD-rGO system in the presence of 1,2-dodecanediol (DDT), a well-known hole scavenger [41,42]. First, a solution of DDT in chloroform was added to the QD-rGO colloidal solution with an rGO/QD ratio of 1:15. The resulting change in the system’s PL properties is shown in Figure 2. In Figure 2a, PL lifetimes increase gradually with the addition of DDT until the average PL lifetime of the system (inset of Figure 2a) reaches saturation with 38 μL of added DDT solution (7 × 10^5^ DDT molecules per QD). The same trend can be observed for the steady-state measurements shown in Figure 2b. DDT addition leads to an increase in QD PL intensity until the integrated PL intensity saturates (inset of Figure 2b). The observed phenomena demonstrate that the excess holes in a QD-rGO system govern the PL response from the QDs.

**Figure 3:**
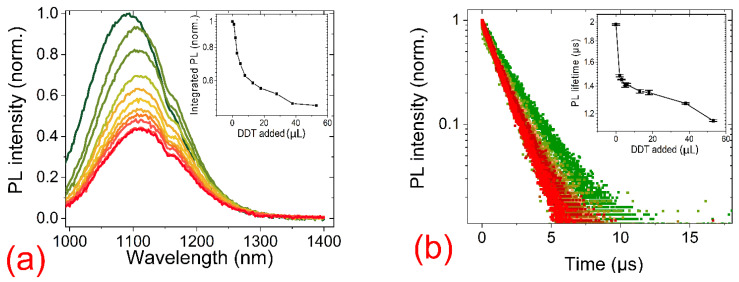
(**a**) PL decay curves for PbS QD solution with different amounts of added DDT (from green = 0 DDT to red = 53 µL DDT). Inset—average PL lifetimes of the QD solution plotted vs. amount of added DDT; (**b**) PL spectra for the QD solution with different amounts of added DDT (from green = 0 DDT to red = 53 µL DDT). Inset—integrated PL intensity plotted vs. amount of added DDT.

In striking contrast to the observed behavior, the addition of DDT to the reference QD solution led to the quenching of both PL intensity and lifetime. The PL spectra quenching and the redshift of PL peak position shown in Figure 3a are in good agreement with previous reports [41,42]. The PL quenching is accompanied by a reduction in PL lifetimes shown in Figure 3b. Thus, DDT in a reference QD solution acts similarly to rGO, causing the extraction of one of the charge carriers. In this case, the uncompensated charge leads to further Auger recombination and a reduction in the intensity and lifetime of the photoluminescence.

## 4. Conclusions

To conclude, we proposed a new model that describes the PL dynamics in rGO/QD systems. This model considers Auger recombination of the excitation induced by excess holes remaining in the QDs after the charge transfer process and non-radiative energy transfer between neighboring QDs. Our model explains the unconventional reduction in PbS QDs PL lifetimes in rGO/QD systems with different component ratios. The critical role of excess holes was confirmed by external engineering of the free carrier density by introducing a hole scavenger to the solutions studied. Removal of excess free carriers leads to a substantial enhancement in PL intensity and growth in PL lifetime. The model proposed here to describe the mechanisms of PL quenching observed in these experiments is valuable for the analysis of the PL properties of 2D/0D systems.

## Figures and Tables

**Table 1:** PL decay curve fitting parameters for QD-rGO systems with various rGO/QD ratios.

Sample	A1	t1, ns	A2	t2, ns	A3	t3, ns	Average PL Lifetime, ns
0:1	0.13 ± 0.01	147 ± 14	0.870 ± 0.003	2212 ± 10			1950 ± 14
1:120	0.38 ± 0.01	62 ± 3	0.26 ± 0.03	840 ± 80	0.35 ± 0.03	2343 ± 88	1080 ± 12
1:60	0.40 ± 0.01	33 ± 2	0.20 ± 0.01	369 ± 20	0.33 ± 0.01	1939 ± 32	750 ± 8
1:40	0.39 ± 0.01	33 ± 1	0.33 ± 0.01	324 ± 10	0.280 ± 0.005	1744 ± 23	610 ± 5
1:30	0.31 ± 0.02	28 ± 2	0.39 ± 0.01	170 ± 8	0.300 ± 0.004	1437 ± 18	510 ± 5
1:20	0.64 ± 0.01	13.0 ± 1	0.280 ± 0.005	123 ± 3	0.080 ± 0.002	1149 ± 23	138 ± 1
1:15	0.51 ± 0.01	4.0 ± 1	0.37 ± 0.01	39 ± 1	0.120 ± 0.003	281 ± 7	51 ± 1

## Data Availability

The data presented in this study are available on request from the corresponding authors.

## Supplementary Materials

The following are available online at https://www.mdpi.com/2079-4991/11/6/1623/s1, Figure S1: The absorption and PL spectra of PbS QDs with 3.6 nm diameter, synthesized for the experiment; Figure S2: FTIR spectra of rGO sheets before (a) and after (b) functionalization; Figure S3: SEM images of rGO sheets; Figure S4: Simulated plot of τ_av vs. rGO/QD ratio assuming that PL quenching is caused by charge transfer only; Figure S5: Plot of τ_av vs. rGO/QD ratio in the rGO-PbS system with 4.8 nm QDs; Figure S6: PL decay curves with fitting curves for QD-rGO systems with various rGO/QD ratios (a,b); the 1:15 QD-rGO system with different amounts of added DDT (c,d); for PbS QD solution with different amounts of added DDT (e,f).

## Appendix A

$k_{A}$ was calculated using these equations as $k_{\mathrm{et}}\to 0$ :

(A1)
$$\tau_{1}=\frac{1}{2k_{r}+k_{\mathrm{nr}}+2k_{A}}\;\to k_{A}=\frac{1-\tau_{1}\left(2k_{r}+k_{\mathrm{nr}}\right)}{2\tau_{1}},\;\tau_{0}=\frac{1}{k_{r}+k_{\mathrm{nr}}},\;Q_{0}=k_{r}\tau_{0}\;\to {\;k}_{r}=\frac{Q_{0}}{\tau_{0}},\;k_{\mathrm{nr}}=\frac{1-Q_{0}}{\tau_{0}}\;\to \;k_{A}=\frac{1-\tau_{1}\left(2\frac{Q_{0}}{\tau_{0}}+\frac{1-Q_{0}}{\tau_{0}}\right)}{2\tau_{1}}$$

where $Q_{0}$ is the initial QD QY.
