# Supplementary material for: Beyond Charge Transfer: The Impact of Auger Recombination and FRET on PL Quenching in an rGO-QDs System
Source: Nanomaterials (Basel). 2021 Jun 21;11(6):1623. doi: 10.3390/nano11061623 (PMC8235269; doi:10.3390/nano11061623)
Supplement: Supplementary file 1 [file nanomaterials-11-01623-s001.zip › nanomaterials-1239978-supplementary.pdf]

nanomaterials

Supporting Information

Beyond Charge Transfer: The Impact of Auger Recombination and FRET on PL Quenching in an rGO-QDs System

Anton A. Babaev [1,*], Anastasiia V. Sokolova [1], Sergei A. Cherevkov [1], Kevin Berwick [2], Alexander V. Baranov [1], Anatoly V. Fedorov [1] and Aleksandr P. Litvin [1]

[1] Center of Information optical technology, ITMO University, 197101 St. Petersburg, Russia; avsokolova@itmo.ru (A.V.S.); s.cherevkov@itmo.ru (S.A.C.); a_v_baranov@itmo.ru (A.V.B.); a_v_fedorov@itmo.ru (A.V.F.); litvin@itmo.ru (A.P.L.)
[2] School of Electrical and Electronic Engineering, TU Dublin, Grangegorman, Dublin 7, Ireland; kevin.berwick@tudublin.ie
* Correspondence: a.a.babaev1@gmail.com

Photoluminescence and absorption spectra of initial QDs measured in tetrachloromethane colloidal solution are presented in Figure S1.

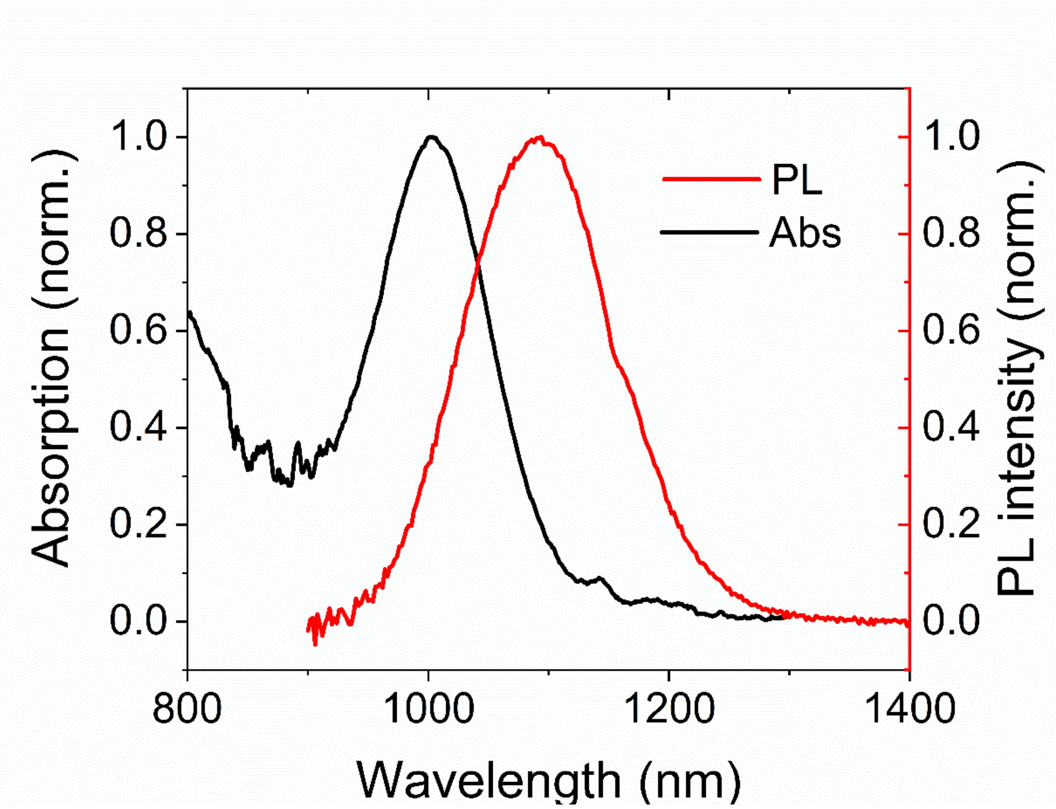

Figure S1. The absorption and PL spectra of PbS QDs with a 3.6 nm diameter, synthesized for the experiment.

Figure S2 shows the rGO FTIR spectra before and after functionalization with 3-mercaptopropyltrimethoxysilane (MPTS) molecules. New peaks for Si–O–C, Si–C, and $–CH_2–CH_2–$ indicate an effective functionalization.

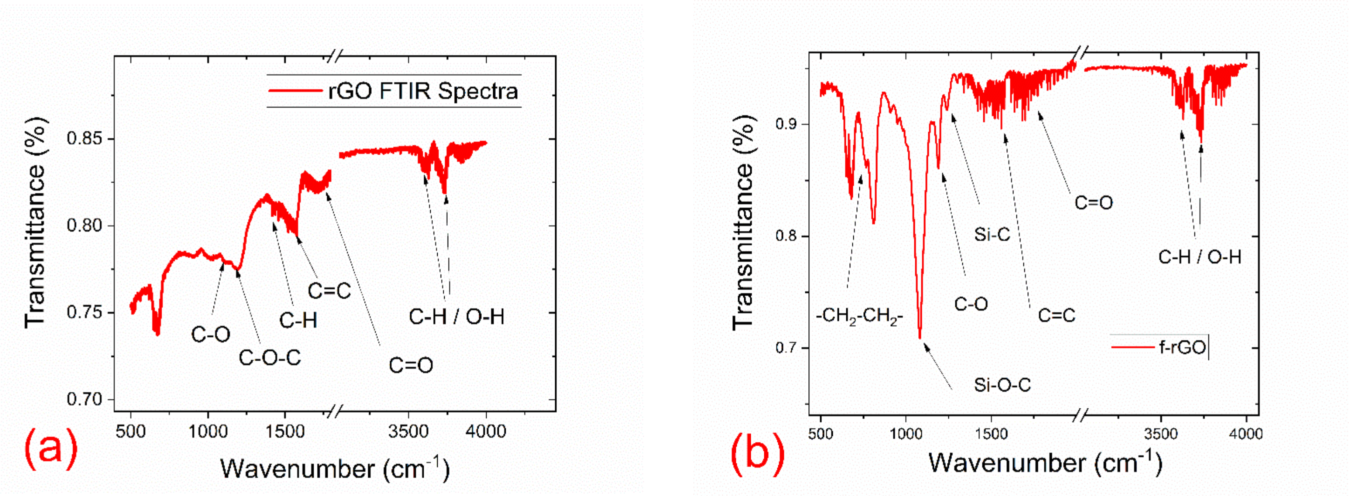

Figure S2. FTIR spectra of rGO sheets before (**a**) and after (**b**) functionalization.

Figure S3 presents the SEM images of the initial rGO. A variety of sheet sizes and shapes can be observed.

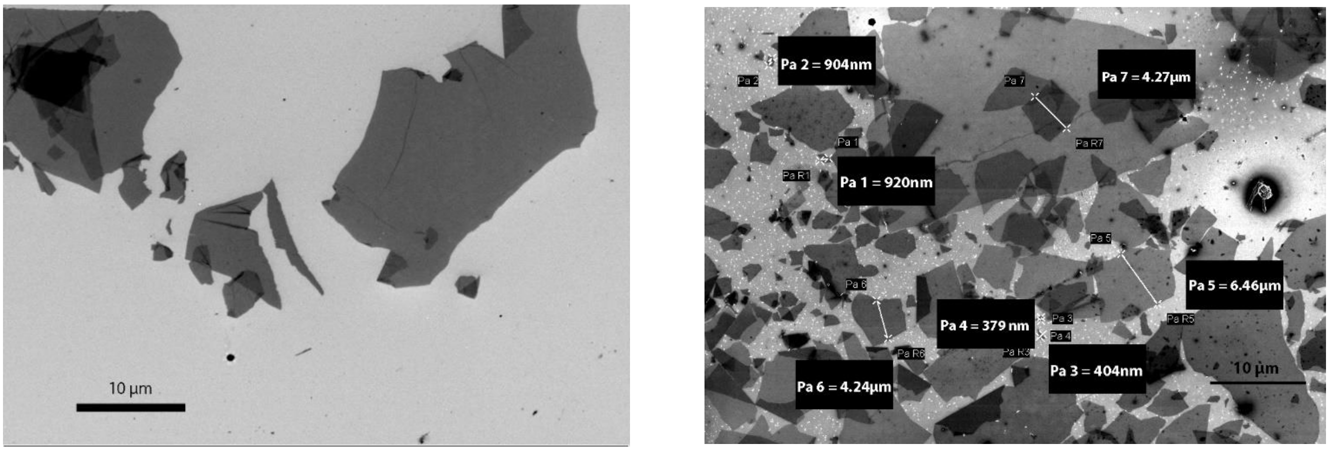

Figure S3. SEM images of rGO sheets.

The τ_{av} to rGO/QD Ratio Plot Simulation

The average PL lifetime in a system with charge transfer induced PL quenching was simulated. As discussed, the only way to observe the dependence of the average PL lifetime τ_{av} to reduced graphene oxide (rGO)/quantum dots (QDs) ratio is to suggest an independent rGO-discharging process. The resulting PL would be a sum of that from quenched and unquenched QDs. Changing the rGO/QD ratio also changes the quenched/unquenched QDs ratio. We simulated the dependence by changing the quantity of unquenched QDs and using the expression below for average PL lifetime:

$$\tau_{av} = \frac{A_0 t_0 + A_q t_q}{A_0 + A_q},$$

where $A_0 = N_0 Q_0$ and $A_q = N_q Q_q$ are the amplitudes of the unquenched and quenched QDs, respectively; N_0 and N_q are the numbers of unquenched and quenched QDs, respectively; Q_0 and Q_q are the quantum yields of unquenched and quenched QDs, respectively; and t_0 and t_q are the PL lifetimes of unquenched and quenched QDs, respectively.

Figure S4 shows the τ_{av} vs. rGO/QD ratio plot, assuming that PL quenching is caused solely by charge transfer, together with experimental results. Clearly, the simulation could not explain the experimental data.

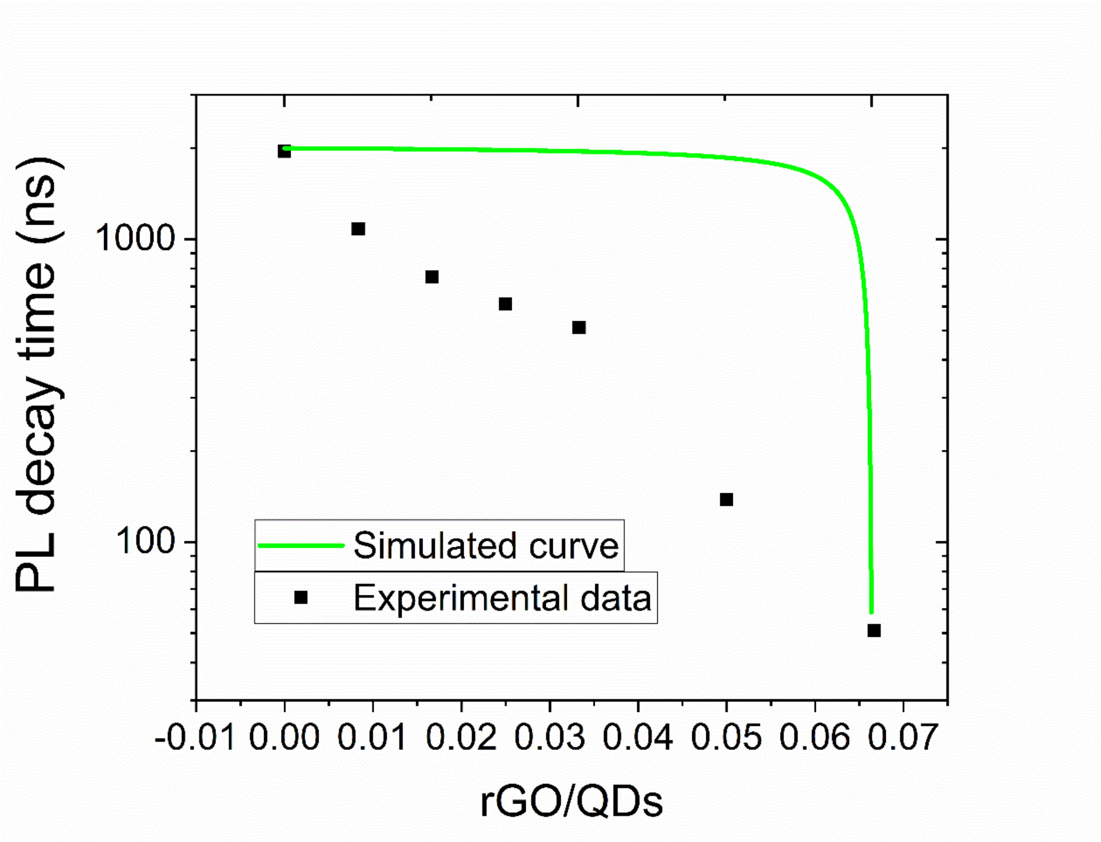

Figure S4. Simulated plot of τ_{av} vs. rGO/QD ratio assuming that PL quenching is caused by charge transfer only.

For a valid calculation of the average PL lifetime of the system with Auger-induced quenched QDs, we required an accurate energy transfer model for the QDs on the rGO sheet. In the observed system, the following dependences can be noted for average inter-dot distance on the rGO sheet (R) and the number of QDs with i and i + 1 excess holes on the rGO sheet (N_{Hi}, N_{Hi+1}): $R \propto N_{rGO}/N_{QDs}$, $N_{Hi+1}/N \propto N_{rGO}/N_{QDs}$.

It is important to note that charged QDs exhibit a 28 meV red shift. Therefore, energy transfer from charged QDs to uncharged (or less charged) QDs is prohibited since the energy shift >kT.

The rGO possesses an aperiodic structure with randomly located functional groups; thus, the QDs positions on the rGO sheet are also randomized. Since the Förster resonance energy transfer (FRET) $\propto R^6$, randomization of QDs positions on the rGO sheet is required.

Simulation of the average PL lifetimes of the rGO-QD system involved the following steps:

(1) The blinking and FRET models were applied for the following calculations. The quantum yield (QY) of the initial QDs and the PL lifetimes of QDs in the H_0 and H_1 states were used to calculate the QDs QY in state Hi. PL lifetimes within the QD in state H_i were determined using the following equations [1]:

$$\tau_{PL}^{(i)} = \frac{1}{k_r^{(i)} + k_{nr} + k_A^{(i)}},$$

$$k_r^{(i)} = n_e n_h k_r,$$

$$k_A^{(i)} = n_e n_h (n_e + n_h - 2) k_A,$$

$$n_h = 1 + i, n_e = 1$$

$$Q_1/Q_0 = 2\left(\tau_{PL}^{1}/\tau_{PL}^{0}\right),$$

$$Q_2/Q_1 = 3/2\left(\tau_{PL}^2/\tau_{PL}^1\right),$$

where n_e and n_h are the number of electrons and holes in the QD; k_r^i is the rate of radiative recombination in the QD with i excess charge carriers; $k_A^{(i)}$ is the rate of Auger-recombination in the QD with i excess charge carriers; $Q_{0,1,2}$ are the PL quantum yields of QDs with zero, one, and two excess charge carriers; $\tau_{PL}^{(i)}$ is the PL lifetime within a QD with i excess charge carriers; $k_r^{(i)}$ and $k_A^{(i)}$, are the radiative and Auger recombination rates in QDs with i excess charge carriers, respectively; k_r and k_{nr} are the radiative and non-radiative recombination rates of the initial QDs, respectively; and k_A is the Auger recombination rate.

The Förster radius, R_F, for the FRET was computed from the Förster model [2] as:

$$R_F^6 = \frac{9000\ \ln 10 k^2 QY_d}{128\pi^5 n^4 N_a}\int I_D(v)\varepsilon_D(v)v^{-4}dv\,,$$

where $\int I_D(v)\varepsilon_D(v)v^{-4}dv$ is the integral of overlap between donor emission $I_D(v)$ and acceptor absorption $\varepsilon_D(v)$ using a wavenumber scale v, k is the orientation factor, n is the medium refractive index, N_a is the Avogadro constant, and QY_d is the donor quantum yield.

The initial distance between the QDs was determined from considerations of QD closely packed at low rGO/QD rates:

$$R_0 = d_{QD} + 2 * r_{shell}$$

in the plane of the sheet and for the QDs on opposite sides of the rGO sheet:

$$R_{0z} = d_{QD} + 2 * r_{MPTS} + \text{rGO thickness}$$

where d_{QD} is the QD diameter, r_{shell} is the oleic acid molecule length, and r_{MPTS} is the MPTS molecule length.

(2) The QDs positions on the rGO sheet are represented in the form of two 100 × 100 arrays in the calculation for the front and back planes of the sheet. The array elements represent the states of the QDs: zero for H_i and 1 for H_{i+1}. Each QD in the array has four attributes: the relative PL intensity, PL lifetime, QY, and a vector of QD location counted from the QD's central position. The central position can be visualized as the geometric center of a square of side R_0 on the rGO sheet. The similar 100 × 100 squares contain QDs.

(3) The number of QDs in the H_{i+1} state is used as the index for iteration. Since $N_{Hi+1}/N \propto N_{rGO}/N_{QDs}$, and N = 20,000 is the total number of QDs, it can be argued that τ_{av} (N_{Hi+1}) will have the same shape as τ_{av} (N_{rGO}/N_{QDs}).

(4) The index for iteration takes values from 0 to N = 20,000. For each value of N_{Hi+1}, the QD arrays are filled with N-N_{Hi+1} zeroes and N_{Hi+1} ones with a relative PL intensity equal to unity, corresponding PL lifetime, QY, and location vectors. The side of the square area occupied by the QD is calculated as: $r = R_0 * \sqrt{(1+\frac{N_1}{N})}$, and the location vector length is limited by the value, $\sqrt{2}\frac{r-d_{QD}-r_{shell}}{2}$. After the creation of the QD arrays and their attributes, the FRET rate is calculated for the nine nearest neighbors to each QD as $k_T(r) = \frac{1}{\tau_D}(\frac{R_F}{r})^6$ where τ_D is the donor PL lifetime. The energy transfer efficiency for each QD can be found according to:

$$E = \frac{k_T(r)}{\tau_D^{-1}+k_T(r)},$$

The value of each QD PL amplitude is reduced by the value of E multiplied by the current QD amplitude value, while the reduction value is transferred to the neighboring QD amplitudes scaled by the $k_T(r)$ value of each neighboring QD. The reduction and increase for each QD are calculated simultaneously for the iteration number equal to $\tau_i \sum_n k_T(r_n)$ average value, where n is the index for the nine neighbors of the QD.

Finally, the average PL lifetime of the system is calculated as:

$$\tau_{av} = \frac{\sum_j \alpha_j \tau_j Q_j}{\sum_j \alpha_j Q_j},$$

where τ_j is the PL lifetime of the QD; α_j is the QD amplitude; and Q_j is the QY of the QD.

The experimental methods used for the 4.8 nm QDs were the same as those used for the main experiment. The resulting average PL lifetimes vs rGO/QDs plot is shown in Figure S5.

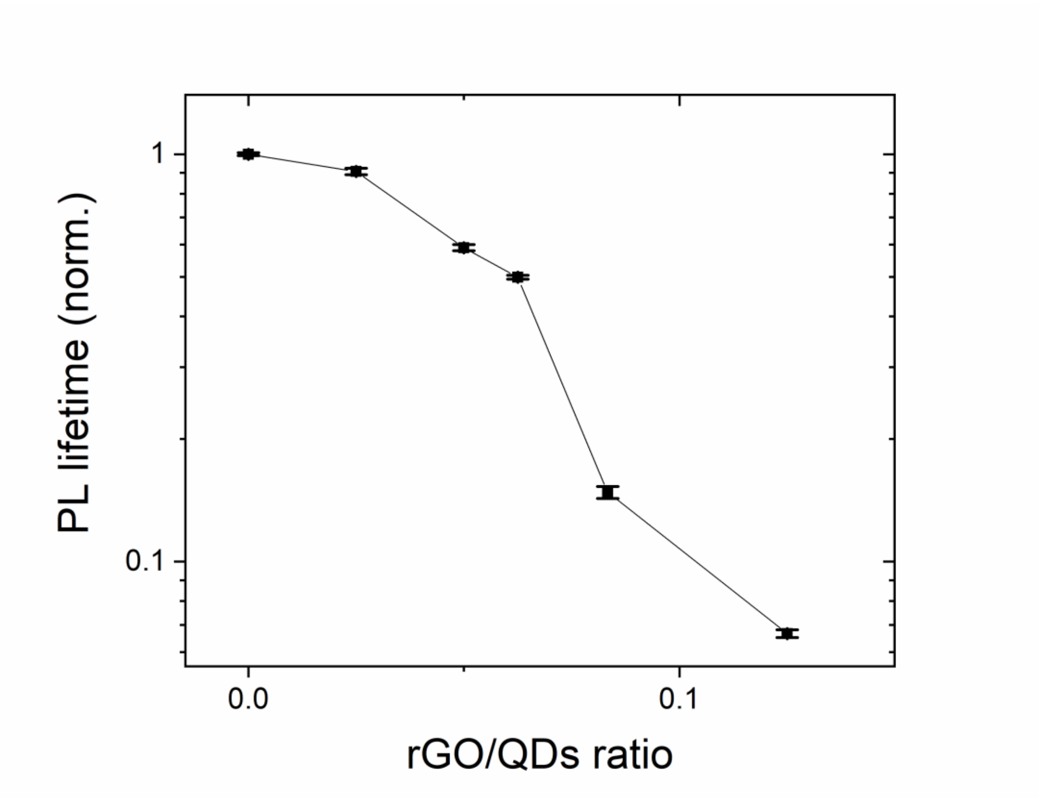

Figure S5. Plot of τ_{av} vs. rGO/QD ratio in the rGO-PbS system with 4.8 nm QDs.

The PL decay curves from Figures 1a, 3a, and 4b of the manuscript are shown with fitting curves in Figure S6.

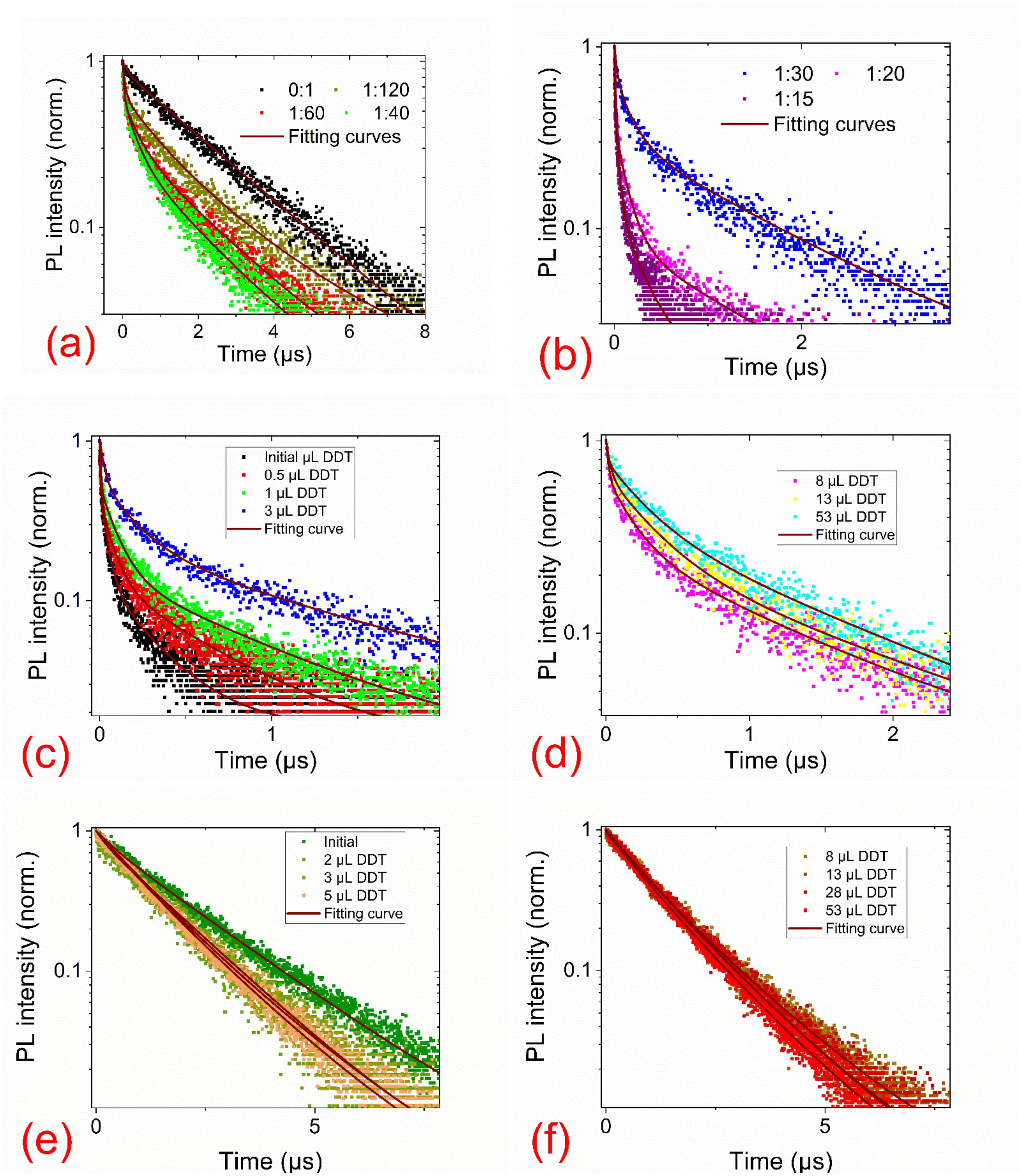

Figure S6. PL decay curves with fitting curves for QD-rGO systems with various rGO/QD ratios (**a**,**b**); the 1:15 QD-rGO system with different amounts of added DDT (**c**,**d**); and for PbS QD solution with different amounts of added DDT (**e**,**f**).

Reference

1. Galland, C.; Ghosh, Y.; Steinbrück, A.; Sykora, M.; Hollingsworth, J.A.; Klimov, V.I.; Htoon, H. Two types of luminescence blinking revealed by spectroelectrochemistry of single quantum dots. *Nature* **2011**, *479*, 203–207, doi:10.1038/nature10569.
2. Lakowicz, J.R. *Principles of Fluorescence Spectroscopy*; Springer: New York, USA, 2006; ISBN 0387312781.
